# Prevalence and Antibiotic Resistance of ESKAPE Pathogens Isolated in the Emergency Department of a Tertiary Care Teaching Hospital in Hungary: A 5-Year Retrospective Survey

**DOI:** 10.3390/antibiotics9090624

**Published:** 2020-09-19

**Authors:** Ria Benkő, Márió Gajdács, Mária Matuz, Gabriella Bodó, Andrea Lázár, Edit Hajdú, Erika Papfalvi, Peter Hannauer, Péter Erdélyi, Zoltán Pető

**Affiliations:** 1 Department of Clinical Pharmacy, Faculty of Pharmacy, University of Szeged, 6725 Szeged, Hungary; matuz.maria@med.u-szeged.hu; 2 Central Pharmacy Department, University of Szeged, Albert Szent-Györgyi Health Center, 6725 Szeged, Hungary; bodo.gabriella@med.u-szeged.hu; 3 Department of Emergency Medicine, University of Szeged, Albert Szent-Györgyi Health Center, 6725 Szeged, Hungary; hannauer.peter@med.u-szeged.hu (P.H.); erdelyi.peter@med.u-szeged.hu (P.E.); peto.zoltan@med.u-szeged.hu (Z.P.); 4 Department of Pharmacodynamics and Biopharmacy, Faculty of Pharmacy, University of Szeged, 6720 Szeged, Hungary; gajdacs.mario@pharm.u-szeged.hu; 5 Institute of Clinical Microbiology, Faculty of Medicine, University of Szeged, 6725 Szeged, Hungary; lazar.andrea@med.u-szeged.hu; 6 Infectious Disease Ward, 1st Department of Internal Medicine, University of Szeged, Albert Szent-Györgyi Medical Center, 6725 Szeged, Hungary; horvathne.hajdu.edit@med.u-szeged.hu (E.H.); papfalvi.erika.piroska@med.u-szeged.hu (E.P.)

**Keywords:** antimicrobial resistance, ESKAPE, emergency department, epidemiology, empirical antibiotic guide, usual drug resistance, UDR, difficult-to-treat resistance, DTR, MDR, XDR, resistance indicators

## Abstract

Antibiotic treatments initiated on Emergency Departments (ED) are empirical. Therefore, knowledge of local susceptibility patterns is important. Despite this, data on expected pathogens and their resistance profile are scarce from EDs internationally. The study aim was to assess the epidemiology and resistance patterns of bacterial isolates from a tertiary-care ED over 5 years, focusing on ESKAPE bacteria (including the Enterobacterales group). After removal of duplicates, *n* = 6887 individual bacterial isolates were recovered, out of which *n* = 4974 (72.22%) were ESKAPE isolates. *E. coli* was the most frequent isolate (2193, 44.1%), followed by the *Klebsiella* genus (664; 13.4%). The third most frequent isolate was *S. aureus* (561, 11.3%). In total, multi-drug resistance (MDR) was present in 23.8% and was most prevalent in *A. baumanii* (65.5%), *P. mirabilis* (42.7%), and *K. pneumoniae* (32.6%). MRSA was isolated in 19.6%, while ESBL-producing Enterobacterales in 17.7%, and these were associated with remarkably higher resistance to other antibacterials as well. Difficult-to-treat resistance (DTR) was detected in 0.5%. The frequent isolation of some ESKAPE bacteria and the detected considerable acquired resistance among ED patients raise concern. The revealed data identified problematic pathogens and will guide us to set up the optimal empiric antibiotic protocol for clinicians.

## 1. Introduction

Antimicrobial resistance (AMR) is a serious threat to public health worldwide. Comprehensive knowledge on the burden and associated outcomes of multi-drug-resistant (MDR) infections is still lacking. Nevertheless, several studies revealed the association between infections caused by antibiotic-resistant bacteria and negative patient outcomes, such as longer hospital stays, higher morbidity and mortality [1,2,3,4]. Infections caused by MDR pathogens are often needed to be treated with more expensive and/or more toxic agents (e.g., colistin, daptomycin) [5,6], which may also affect patient outcomes. The financial burden of AMR is also considerable [2,7], one case of infection caused by an AMR pathogen (e.g., an MRSA blood stream infection) can cost up to 20,000 US dollars [2]. AMR is an increasing problem not only in hospitals, but also in community-acquired infections, associated with the successful penetration and spread of MDR clones from nosocomial settings [8]. 

Based on overall mortality and economic impact, the so-called “ESKAPE” group of pathogens deserve the utmost attention, both from clinical and from research and development aspect [9,10]. Rice introduced the ESKAPE acronym in 2008 for the group of pathogens capable of ‘escaping’ the biocidal action of antibiotics, namely E: *Enterococcus faecium*, S: *Staphylococcus aureus*, K: *Klebsiella pneumoniae*, A: *Acinetobacter baumannii*, P: *Pseudomonas aeruginosa*, E: *Enterobacter* spp [11]. These pathogens rank among the most prevalent causes of life-threatening infections, and their ability to acquire and disseminate antimicrobial resistance is well known [12,13]. Recently, the World Health Organisation (WHO) developed a global priority list of antibiotic-resistant pathogens to guide new antibiotic discovery, where certain antibiotic resistance in the Enterobacterales family is listed among the critical priorities (e.g., third generation cephalosporin-resistant Enterobacterales) [14]. Hence, in some publications, the entire Enterobacterales group is often added to the ESKAPE group of bacteria [15,16] and the letter ‘S’ may be complemented by *Stenotrophomonas maltophilia*, as that has emerged as global opportunistic human Gram-negative pathogen [17]. 

One of the main drivers behind the development of AMR is the misuse and overuse of antibacterial agents [18,19]. As the utilisation of antibacterials can be controlled [20], rationalization of use is of pivotal importance to curb AMR. Emergency care departments (EDs) are unique in the health care system, providing an interface between ambulatory care and hospital care settings [21]. Physicians work at ED under substantial time-pressure [22]. Moreover, in contrast to General Practitioners, ED physicians should make decisions on antibiotic prescribing based on limited background information on the patient. With high rates of patient turn-over, variability in staffing and a subsequent lack of ‘continuity of care’ are important challenges in facilitating prudent use of antibiotics [23]. Out-of-hour services and EDs has been associated with lower threshold for antibiotic prescribing and suboptimal antibiotic choice [24,25,26,27,28]. Antibiotic treatments initiated on EDs are almost exclusively empirical. Therefore, the knowledge of local susceptibility patterns is of crucial importance in the antimicrobial stewardship [21,29]. Moreover, research has found that both isolated bacteria and related antibiotic susceptibility patterns differ between isolates originating from the ED and cultures obtained in other hospitalized patients, hence general hospital resistance maps may not serve as optimal guide for empirical treatment initiated at ED [30]. Despite this, data on expected pathogens and their resistance profile in community-acquired infections [31] and specifically data from ED are scarce.

Therefore, the aim of the present study was to assess the epidemiology and resistance patterns of bacterial isolates from the ED that may help to identify problematic pathogens and to set up an optimal empiric antibiotic guide for clinicians.

## 2. Results

### 2.1. Distribution of Bacterial Isolates

During the study period, *n* = 6887 individual bacterial isolates were recovered from clinical specimens, out of which *n* = 4974 (72.22%) were ESKAPE pathogens. Overall, as shown in Table 1, we observed the dominance of Gram-negative bacteria. The Enterobacterales group was predominant among all ESKAPE isolates, with 75% of all isolates being from this group. *Escherichia coli* was the most frequently isolated bacteria (2193, 44.1%), followed by the *Klebsiella* genus (664; 13.4%), most frequently *K. pneumoniae*. The third most frequent isolate was *S. aureus* (561, 11.3%), followed by *Proteus* spp. (526, 10.57%). *Stenotrophomonas maltophilia, A. baumannii*, and the *Providencia*, *Salmonella* and *Serratia* genus played limited role, with below 1% of share within the ESKAPE group.

The most frequent clinical specimens were blood cultures, urinary catheter or midstream urine specimens, and deep wound or abscess samples (Table 2). Respiratory samples were only taken rarely (*n* = 121) mainly from critically ill patients. We observed the dominance of *Escherichia* isolates in blood and urine samples. In blood cultures, *S. aureus* was the second most frequent isolate, whereas in urine the *Klebsiella* genus. In wound/abscess specimens *S. aureus* were isolated most often, followed by the *Proteus* spp.

### 2.2. Bacterial Resistance According to Specific Indicators

**Figure 1:**
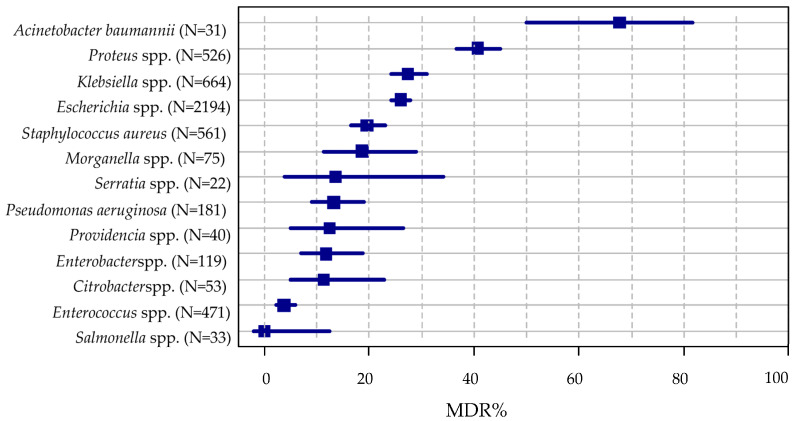
Rate of multi-drug resistance among ESKAPE isolates.

The overall resistance level of the ESKAPE isolates is summarized in Table 3. In total more than half of the isolates (55.31%) possessed intrinsic resistance only (i.e., wild type), however these ranged between 12.9%% (*A. bauminii*) and 84.85%% (Salmonella genus) among different isolates. Usual drug resistance (UDR) was developed in 44.69%% of isolates, and ranged between 15.15%% (*Salmonella* spp.) and 87.10%% (*A. bauminii*). UDR was prevalent among the most frequently isolated bacteria (i.e., *E. coli* and *Klebsiella* spp.). Multi-drug resistance (MDR) was acquired in almost every fourth isolate and showed considerable range between different ESKAPE isolates (Figure 1). MDR was most prevalent in *A. bauminii*, followed by *Proteus*, *Klebsiella*, and *Escherichia* spp. and lowest in Salmonella spp. For the most frequently isolated Gram-positive bacteria, *S. aureus*, the prevalence of MDR was nearly 20%. For the Enterococcus genus MDR was detected on average in 3.83%%, but species-level data showed extreme diversity (see below). DTR was present in 23 isolates (among which, *Klebsiella* spp., *A. baumannii*, *P. aeruginosa*, and *Proteus* spp. were implicated) while we detected only one pandrug-resistant *P. aeruginosa* isolate.

### 2.3. Bacterial Resistance to Specific Antibacterials

Table 4 (for Gram-positive bacteria) and Table 5 (for Gram-negative bacteria) show the susceptibility rate of ESKAPE isolates to specific antibiotics and MDR rates at species-level. *E. faecalis* species can be effectively treated with first line agent such as ampicillin, as all isolates proved to be susceptible. In contrast, in case of *E. faecium* isolates, the clinical success can be anticipated in case of tigecycline and linezolid, which are both reserve antibacterial agents based on in-vitro data. Vancomycin resistance among *E. faecium* was detected in every third case, while MDR can be encountered in nearly every second isolate (45.5%). Methicilin-resistant *S. aureus* (MRSA) were isolated in 19.6% (95/561). Over the years, no clear increasing or decreasing trend for MRSA has been observed (data not shown). Methicillin-resistant strains (MRSA) were also considerably less susceptible to many other antibacterial agents (see Table 4) compared to methicillin-susceptible strains (MSSA). Effective treatment for MRSA can be anticipated by sulfamethoxazol-trimethoprim or glycopeptides.

Different *Klebsiella* species showed different resistance levels to certain antibacterials (Table 5). *K. pneumoniae* isolates had low susceptibility rate (below 70% or 80%) to all beta-lactams except carbapenems (indicative of ESBL production) and presence of MDR in every third isolate (32.6%). *A. baumannii* isolates showed high resistance level to all tested antibacterials, except the reserve antibiotic colistin to which all tested organisms (*n* = 15) were fully susceptible. *P. aeruginosa* strains were highly susceptible to ceftazidime (90.6%), cefepime (91.7%), and aminoglycosides (over 90%) and clinical success can be anticipated by also piperacilin-tazobactam empirical treatment, as overall susceptibility rate was 87.2%. Out of the four *P. aueruginosa* isolates that were tested for colistin, one isolate, considered extensively drug-resistant (XDR) was resistant to colistin. 

*E. coli*, the most frequently isolated Gram-negative bacteria were susceptible to aminoglycosides in ~90% (see Table 5). Co-amoxiclav, cefuroxime, and third generation cephalosporin susceptibility among *E. coli* isolates ranged between 80% and 90%. Based on the susceptibility rates, the treatment of infections caused by *P. mirabilis* are cumbersome (susceptibility to different beta lactam agents ranged between 40.4% and 75.5%, while ciprofloxacin susceptibility was 57.7%), while *P. vulgaris* strains were highly susceptible (over 90%) to many beta-lactam agents (see Table 5). ESBL-positive Enterobacterales strains (659/3719; 17.7%) were generally less susceptible to non-beta lactam antibacterial agents compared to ESBL-negative strains. Concerning the whole Enterobacterales group, no clear temporal trend in the rate of ESBL-positive strains has been observed (ranged between 14.6% and 20.5%, data not shown). 

## 3. Discussion

The present study reveals the bacterial spectra and susceptibility patterns of ESKAPE pathogens in a level I ED in Hungary. As such data from Hungary has not been published before, the work fills in an important gap in knowledge. Moreover, epidemiological reports from EDs are also scarce internationally [30,31,32,33,34,35,36,37]. As most of these studies focus on certain infections or specimen types [31,32,33,34,37] or only on certain bacteria or bacterial families [34,35], the possibility of meaningful benchmarking is often limited.

However, at some point, we referred to the latest report of the European Surveillance of Antimicrobial Resistance network (EARS-NET) [38], which compare antibiotic susceptibility data of certain bacterial isolates (from invasive samples) across the member states, hence providing up-to-date knowledge on resistance levels.

In this study, the most common clinical specimen was catheter-specimen urine, followed by blood culture samples. ESKAPE organisms were responsible for 72.22% (4974/6887) of all positive bacterial clinical isolates, and we detected the predominance of Gram-negative bacteria. The most common isolates were from the Enterobacterales family, and within the group *E. coli* was the most frequently isolated organism in urine and blood samples, while *S. aureus* and the *Klebsiella* genus was the second most frequent organism isolated from blood culture and urine samples, respectively. A study from a US emergency department, that similarly to our study, focused on all clinical specimens, also revealed the dominance of Gram-negative bacteria and especially *E. coli* [30]. A survey from a German ED [31] found the Enterobacterales group as the most commonly isolated pathogens in cases of true bacteremia (59.3%), followed by *S. aureus* and this pathogen spectra has been found in another ED study focusing on blood stream infections (BSI) [32]. *E. coli* and *Klebsiella* spp. were the most frequent isolate in other ED studies focusing on urinary tract infections (UTIs) [36,39].

We also isolated *Strenotrophomonas maltophilia* and *A. baumannii* isolates from the ED patients; this phenomenon is concerning, as these bacteria are predominantly considered as hospital-acquired pathogens, often associated with infections in immunocompromised individuals. The isolation of *Strenotrophomonas maltophilia* and *Acinetobacter* spp. was reported only from one epidemiological study from EDs [30]. In an Italian hospital-based study focusing on ESKAPE pathogens in BSI, *Acinetobacter* spp. was not involved in any community-acquired infections but was prevalent in hospital acquired BISs [40]. 

**Figure 2:**
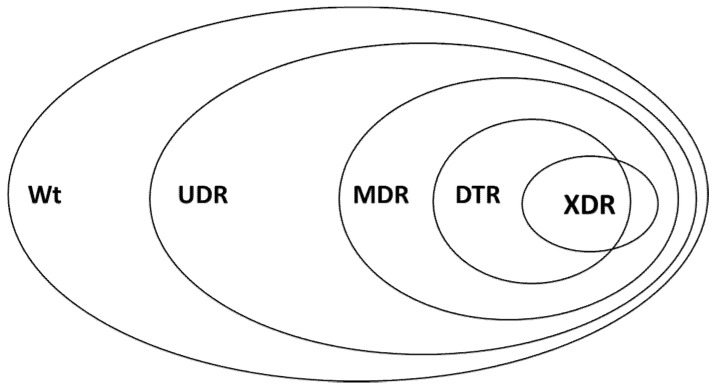
Visual representation of the relationship of various resistance-categories used in this study Wt: wild-type/susceptible; UDR: usual drug resistance; MDR: multi-drug resistance; XDR: extensive drug resistance; DTR: difficult-to-treat resistance; PDR: pandrug-resistance.

Existing resistance classifications (MDR-XDR-PDR) have been used worldwide and they have been praised in their utility for microbiological and epidemiological purposes [41]. Nevertheless, many studies have pointed out that this classification system of bacteria may not correlate well with clinical outcomes. Therefore novel resistance indicators has been introduced in the last years: UDR and DTR (see definitions in the methods). As of recently, bacterial resistance may be characterized into 5 resistance categories, and these categories represent a continuum (wild-type>>UDR>>MDR>>DTR>>XDR>>PDR) (see Figure 2), reflecting on the ease of effective antibacterial therapy selection. In this study, 55.3% of the bacterial isolates were wild type, meaning that every second isolate possessed acquired resistance (i.e., 44.7% of isolates were considered to have usual drug resistance-UDR). DTR resistance was detected among 0.46% of Gram-negative organisms, but this ranged widely by taxon (*A. baumanii*: 41.94%, *Klebsiella* spp.: 0.15%, and no DTR in *E. coli*). Regarding the MDR category, 23.8% of the bacterial isolates were classified as MDR, and highest rates were observed for *A. baumannii, P. mirabilis, K. pneumoniae* and *E. coli*, respectively (Figure 1). 

No published ED studies has reported the prevalence of UDR, DTR or even MDR, so we limited our comparison to some hospital-based studies. 

Two multicenter studies on bloodstream infection from US hospitals found DTR in 1% and 1.3% of isolates respectively [42,43], and, similarly to our data, it was highest in *A. baumanni* (18.3% and 17.2%) and lowest in *E. coli* similar to our study (0.04% and 0.07%). In a Korean multicenter study [44] on Gram-negative BSIs, significantly higher rate of isolates were classified as DTR (12.6%), no DTR *E. coli* was found, and DTR was highest in *A. baumannii* (79.6%) [44]. However, this Korean study was limited to only four taxa (*E. coli, K. pneumoniae, P. aeruginosa*, and *Acinetobacter* species, so any comparisons should be very cautiously made. Gianella et al. [45] assessed the epidemiology and resistance characteristics of Gram-negative BSIs in Bologna, Italy; and observed the following resistance rates: MDR: 21.9% (vs. 23.8% in our study), DTR: 11.0% (vs. 0.46% in our study). The overall MDR rate in Gram-negative bacilli has been reported 10.67% at a tertiary care internal medicine unit in Romania [46]. Regarding non-fermenting Gram-negative bacteria, an Italian single-center hospital-based study, the MDR rates of *P. aeruginosa* was 34.8% in community-acquired infections (vs. 13.3% in this study), while no *Acinetobacter* spp. was isolated from community-acquired infections. The MDR rate of the Enterobacterales group was nearly 100% in our study, while it was 40.8 in ESBL positive *E. coli* and 78.8% in ESBL positive *K. pneumoniae*.

Since the millennium, several reports showed a substantial increase of Enterobacterales-producing ESBLs and carbapenemases worldwide [38,47,48,49]). In our study ESBL positive Enterobacterales was 17.7%. At species-level, 31% of the *K. pneumoniae* were ESBL producers (i.e., ceftriaxone susceptibility was 69%). while *E. coli* isolates were ESBL producers in 14%. In a recent German ED study [31] ceftriaxone non-susceptibility among Enterobacterales in BSI was 11.1%. In the US, a single center ED study focusing on UTI [37] reported ESBL positive *E. coli* rate of 5.9%, while in a Taiwan ED study revealed ESBL positivity in 1.7% of *E. coli* and in 0.9% of *K. pneumoniae* isolated from blood. Ceftriaxone resistance was reported at 10% in urinary *E. coli* isolates [36] from a US Emergency Department. In the EARS-NET report, the EU mean of third generation cephalosporin-resistant *E. coli* in invasive isolates was 15.1%, and this rate was 31.7% for *K. pneumoniae*. All these confirm that ESBL-producing Gram-negative pathogens are prevalent in our ED and that this organism has been expanded to the community setting. The study of Liu et al. confirmed that ESBP-producing isolates were more prevalent in nursing home residents admitted to the ED [35]. As nursing home residents are often admitted to our ED, this might partly explain the high ESBL rate at our unit. 

An Italian study also confirmed the presence of ‘problematic’ pathogens in the outpatient setting: they have found no significant difference in the rate of ESBL-producer phenotypes in *E. coli* and *K. pneumoniae* isolates from hospital or community onset BSIs [40]. The presence and dissemination of ESBL-producer strains are important challenge as therapeutic options for these organisms are limited. Additionally, infections caused by ESBL *E. coli* and *K. pneumoniae* are associated with increased mortality and length of hospital stay or worse clinical outcome compared to non-ESBL-producing isolates [50,51]. Regarding carbapenem resistance, we have detected only one *K. pneumoniae* isolate resistant to meropenem, while all other Enterobacterales species were fully susceptible to carbapenems except one *P. mirabilis* isolate that were resistant to imipenem and ertapenem. However, if the spread of ESBLs will not be contained in our setting, and the use of carbapenems will not be controlled, which may favor the selection of carbapenemase-producing Enterobacterales in the future. Similar carbapenem susceptibility rates among Enterobacterales were reported from other EDs [31,35,37].

Gentamicin susceptibility of *E. coli* isolates was 89% in the ED study of Jorgensen et al. [36], and the study of Liu et al. from Taiwan [35] reported high amikacin susceptibility (above 90%) in the Enterobacterales group in the isolates of ED patients. These results are comparable to the *E. coli* resistance rate in this study and similar to the EU mean of aminoglycoside resistance in *E. coli* (11.1%) However, for *K. pneumoniae*, we recorded lower (<80%) gentamicin and amikacin resistance rates, which data correspond well to the data of the ESAC-Net report [38]. Considerably higher aminoglycoside susceptibility rate in ESBL-positive isolates compared to ESBL negative isolates were found in our study, similar to the ED study of Liu et al. [35]. The fluoroquinolone resistance of the Enterobacterales group was above 30% in this study, and at species-level, it was even higher in common pathogens like *K. pneumoniae* and *P. mirabilis*. Ciprofloxacin resistance was 22.9% in the Enterobacterales group in the German ED [31], and *E. coli* resistance ranged between 23% and 25.2% in American EDs [34,36]. Similarly, high ciprofloxacin resistance (44.9%) for the Enterobacterales group were only reported from Taiwan (Liu et al.) [35]. The high ciprofloxacin resistance among Gram-negative urinary isolates from outpatients has been previously reported for the university central microbiological laboratory [52]. The high fluoroquinolones resistance level is the direct consequence of the exceptionally high relative use of fluoroquinolones in Hungary both in the ambulatory care and in the hospital setting [53]. All these results confirm that fluoroquinolones empiric therapy should be avoided in the ED setting. 

*A. baumannii* and *P. aeruginosa* are both opportunistic pathogens associated primarily with healthcare-associated infections. Both species are intrinsically resistant to many antimicrobial agents. In this study, only colistin, a “reserve antibiotic”, showed to be effective *in-vitro* for all tested *A. baumannii* isolates. Only 55.2% of the *A. baumannii* isolates were susceptible to meropenem. This value is identical to the reported value for Hungary and considerable higher than the European average (31.9% in 2018) in the latest EARS-NET surveillance system report [38], which, in contrast to our study, encompass only invasive isolates. 

Considering *P. aeruginosa* isolates in this study, effective antibacterial therapy may be anticipated in most cases using anti-pseudomonal beta-lactam agents. Interestingly, meropenem susceptibility of *P. aeruginosa* was slightly lower than susceptibility to third generation cephalosporins. The presence of carbapenem-resistant but cephalosporin-susceptible (Car-R/Ceph-S) *P. aeruginosa* strains has been already reported from our university [54]. 

However, ciprofloxacin resistance rate exceeded 20% (i.e., susceptibility was 77.9%), and this was slightly above the EU mean revealed by the European Surveillance [38]. A recent study focusing on a single German ED found similar ciprofloxacin susceptibility rate (81.3%) for *P. aeruginosa* recovered from blood stream infections [31]. In our study, gentamicin susceptibility was 90.1%, and amikacin susceptibility was 96.7% in *P. aeruginosa* isolates. Gentamicin susceptibility was higher (96%) in *P. aeruginosa* in the ED study of Draper et al. [30], while no aminoglycoside susceptibility was reported from the study of Rothe et al. [31].

Among *S. aureus* isolates, 16.9% were identified as MRSA with no identifiable temporal trends. This is identical to the EU mean (16.4%) reported in 2018 and slightly lower than the Hungarian average (23.1%) reported by the EARS-Net. In comparison, Kao et al. from Taiwan [32] reported MRSA rate of 6.8%, Rothe et al. [31] reported a rate of 3.3% in ED isolates. Draper et al. [30] found similar MRSA rate (19.6%) at their ED, which was slightly lower than reported for the entire hospital. The practical consideration of MRSA isolates is the fact that resistance to other antibacterials occurred at higher rate compared to MSSA isolates. 

Regarding *Enterococcus* spp., no vancomycin-resistant *E. faecalis* isolates were detected, while vancomycin susceptible *E. faecium* was only 66.7% (resistance rate 33.3%). This rate is much higher that the EU mean resistance of *E. faecium* to vancomycin (17.3%) and similar to the value reported for Hungary (39.5%) [38]. In an Italian hospital-based study, out of the 1628 isolates from community acquired infections, n = 19 was *E. faecium* and vancomycin-resistance was detected in only 1 case, corresponding to 5.3% [40]. From a recent German ED study, no VRE was isolated [31]. All these means that if the pathogenic role of *E. faecium* is suspected, one should consider the possibility of glycopeptide-resistance. 

### Strengths and Limitations

The present study provides the landscape of ESKAPE pathogens causing a variety of infections in an ED over a 5-year surveillance period to serve as a basis for antimicrobial stewardship interventions. Such an epidemiological snapshot is scarce in the literature for emergency departments, and has not been reported from Hungary. Also, this is one of the first studies that use the novel resistance indicators (i.e., DTR) in other than an ICU setting and other than blood stream infections.

We have to acknowledge the limitations of this research. This is a single-center study based on a retrospective analysis of laboratory data without linkage to clinical outcome or to patient characteristics. However, the heterogeneous patient population of this ED, the large catchment area and high patient-turnover may allow some generalization to other EDs. Prior hospitalization is a well-known risk factor for infection/colonization with drug-resistant bacteria. As some of the patients may have been hospitalized before ED presentation or live in long-term care facility, means that our data do not reflect the true prevalence (i.e., might overestimate) the true prevalence of bacteria and AMR in the community. Also, it has been reported that culture sending may be biased by high risk cases and treatment failures [21]. On the other hand, the high prevalence of ESKAPE bacteria among ED patients is alarming, as it proves that the dissemination of AMR bacteria in the community has already occurred.

## 4. Materials and Methods

### 4.1. Study Site, Data Collection and Management, Inclusion Criteria

The present retrospective study was carried out during the time period between 1st of June 2014 and 31st of July 2019 at the central Emergency Department (ED) of the Albert Szent-Györgyi Clinical Center, a tertiary-care teaching hospital (with approx. 1800 beds), in Southern Hungary. 

The level I ED of the University of Szeged has opened on the 1st of May, 2014. The ED has annual census of nearly 40,000 adult patient visits for mainly traumatology, internal medicine, and neurological cases. Due to geographical distances, emergency cases of gynecology, urology, ear-nose-throat, and ophthalmology are provided at the dedicated clinics. During the study, all positive microbiological specimens and data collected at the central ED were retrieved. Data collection pertaining to isolates and records of “ESKAPE” bacteria were performed electronically, in the records of the MedBakter laboratory information system (LIS) by the authors. Non-ESKAPE bacteria and contaminants were excluded from the study. In addition, bacteria of the normal skin flora were considered pathogenic bacteria only if the same bacteria, has been recovered from 2 or more blood cultures with the same antibiotic-susceptibility patterns.

During data collection and analysis, only the first isolate per patient was included in the study; however, isolates with different antibiotic-susceptibility patterns from the same patient or isolation of similar bacteria >7 days apart were considered as different individual isolates. If the same bacteria with identical antibiotic-susceptibility patterns have been isolated from both the urine culture and the blood culture of the same patient on the same day, the urine specimen was removed from the analysis. In addition, anorectal swabs, throat swabs, and vulva specimens (*n* = 16) taken for screening purposes specimens positive for fungal isolates only (*n* = 305) and faeces samples (*n* = 785) were also excluded. 

### 4.2. Laboratory Procedures, Microbial Identification

Processing of clinical samples submitted by the ED to the Institute of Clinical Microbiology was carried out according to routine cultivation and identification guidelines of clinical bacteriology, briefly described below [55]. All samples were processed within 1 h of sampling. Blood culture samples, respiratory tract samples, cerebrospinal fluid samples, and other invasive sample types were subject to Gram-staining to assess for the presence of microorganisms and while blood cells (where relevant). 10 µL of each un-centrifuged urine sample was cultured on UriSelect chromogenic agar plates (Bio-Rad, Berkeley, CA, USA) with a calibrated loop, according to the manufacturer’s instructions and incubated at 37 °C for 24–48 h, aerobically. If the relevant pathogens presented in significant colony count (10^5^ < colony-forming units/mL; however, this was subject to the interpretation of the clinical microbiologist), the plates were passed on for further processing. Aerobic and anaerobic blood culture bottles were incubated in the BacT/ALERT 3D (bioMérieux, Marcy-l’Étoile, France) detection system; blood culture bottles were incubated for 5 days (21 days, if endocarditis was suspected). Positive blood cultures, respiratory tract samples, cerebrospinal fluid samples, and other invasive sample types were routinely processed on blood agar, chocolate agar, eosine-methylene blue agar and Sabouraud agar (purchased from bioMérieux, Marcy-l’Étoile, France); bacterial culture media were incubated at 37 °C for 24–48 h, aerobically, while Sabouraud agar was incubated at 37 °C for 24 h and for additional 6 days at room temperature. Positive blood cultures, cerebrospinal fluid samples and other invasive sample types were also processed in Brain Heart Infusion broth (Oxoid, Basingstoke, UK) and on plated on Schaedler agar (bioMerieux, Marcy l’Etoile, France) containing horse blood 5% *v/v*, haemin, and Vitamin K_1_, for the isolation of anaerobic organisms (not included in the data analysis). The cultivation of anaerobic bacteria was carried out in line with principles in anaerobic bacteriology, at 37 °C in an anaerobic chamber (Baker Ruskinn, York, UK; in an atmosphere of 90% N_2_, 5% H_2_, and 5% CO_2_) for at least 48 h.

Bacterial identification was carried out using the VITEK 2 Compact ID/AST (bioMérieux, Marcy-l’Étoile, France), based on the manufacturer’s recommendations and matrix-assisted laser desorption/ionization time-of-flight mass spectrometry (MALDI-TOF MS). For MALDI-TOF analysis, bacterial cells were transferred to a stainless-steel target with a sterile toothpick, after cultivation of the respective isolates on appropriate culture media, described previously. An on-target extraction was performed by adding 1 µL of 70% formic acid prior to the matrix. After drying at ambient temperature, the cells were covered with 1 µL matrix (α-cyano-4-hydroxy cinnamic acid in 50% acetonitrile/2.5% trifluoro-acetic acid). Mass spectrometry measurements were performed by the Microflex MALDI Biotyper (Bruker Daltonics, Bremen, Germany) in positive linear mode across the m/z range of 2 to 20 kDa; for each spectrum, 240 laser shots at 60 Hz in groups of 40 shots per sampling area were collected. The MALDI Biotyper RTC 3.1 software (Bruker Daltonics, Bremen, Germany) and the MALDI Biotyper Library 3.1 were used for spectrum analysis [56]. Logarithmic (log) score values (ranging between 0–3.0) were determined by the software, by automatically calculating the proportion of matching peaks and peak intensities between the test spectrum and the reference spectra available in the database. log score values 0-1.699 were considered as ‘not reliable identification’, 1.70–2.299 were reported as ‘probable genus-level identification’, while a score ≥2.300 was considered as reliable species-level identification [57]. 16S RNA sequencing was not utilized for identification or otherwise in the Institute during the study period. 

### 4.3. Antimicrobial Susceptibility Testing

Antimicrobial susceptibility testing for the relevant bacterial isolates were carried out based on the methodological recommendations and standards of the European Committee on Antimicrobial Susceptibility Testing (EUCAST) valid at the time of the assays. The Kirby–Bauer disk diffusion method (Liofilchem, Abruzzo, Italy) and gradient tests (Liofilchem, Abruzzo, Italy) were performed on Mueller–Hinton agar (MHA). Colistin susceptibility was performed using the broth microdilution method in a cation-adjusted Mueller-Hinton broth. Susceptibilities to carbapenems and colistin were evaluated using commercial panels (MERLIN Diagnostica, Bornheim, Germany), using cation-adjusted Mueller–Hinton broth. In addition, for the verification of discrepant results, the VITEK 2 Compact ID/AST (bioMérieux, Marcy-l’Étoile, France) was also utilized. The interpretation of susceptibility results was performed based on EUCAST breakpoints valid at the time of the interpretation. Intermediate results were grouped with and reported as resistant. *S. aureus* ATCC 29213, *E. faecalis* ATCC 29212, *P. mirabilis* ATCC 35659, *E. coli* ATCC 25922, *P. aeruginosa* ATCC 27853, *A. baumannii* ATCC 19606 and *S. maltophilia* ATCC 13637 were used as quality control strains.

Methicillin-resistant *S. aureus* (MRSA) strains were detected using mannitol salt agar (MSA) and cefoxitin (FOX) disks (<22 mm zone diameter) and PBP2’ Latex Agglutination Test Kit (Thermo Fisher Scientific Hungary GmbH, Budapest, Hungary) [56]. If extended-spectrum beta-lactamase (ESBL)-production was suspected [58], detection was carried out based on EUCAST recommendations using AmpC-ESBL Detection Set (MAST Diagnostica GmbH, Reinfeld, Germany) and VITEK 2 Compact ID/AST (bioMérieux, Marcy-l’Étoile, France), according to the manufacturer’s instructions. Vancomycin-resistance and carbapenem-resistance was suspected in case of reduced susceptibility or resistance to the relevant agents during routine susceptibility tests; these isolates were sent to the national reference laboratory (at the National Public Health Institute) for further processing.

Susceptibility testing included all relevant antibiotics for the respective bacteria, to allow for their classification into resistance categories based on the criteria detailed in Section 4.4; an antibiotic was not tested and was excluded from the analysis if intrinsic resistance was present, based on the EUCAST Expert Rules on Intrinsic Resistance and Exceptional Phenotypes.

### 4.4. Definitions for the Classification of Resistance Groups

Based on the analysis of the susceptibility testing results, respective isolates were classified into various resistance categories. An isolate was considered as wild-type (Wt) or pan-susceptible, if they were susceptible to all tested antibiotics, excluding those where intrinsic non-susceptibility is present. Isolates were classified in the usual drug resistance (UDR) category, if resistance was observed to at least one agent outside of the group of antibiotics affected by intrinsic non-susceptibility [59]. Multi-drug-resistant (MDR), extensively drug-resistant (XDR) and pandrug-resistant (PDR) bacteria were defined according to the European Centre for Disease Prevention and Control (ECDC) and the US Centers for Disease Control and Prevention (CDC) Expert Proposal and it was matched and updated according to the newest EUCAST Expert Rules on Intrinsic Resistance and Exceptional Phenotypes [41,60]. Briefly, nonsusceptibility to ≥1 agent in ≥3 antimicrobial categories were defined as MDR, susceptibility limited to ≤2 categories were defined as XDR and resistance to all antibiotics was defined as PDR [41]. The difficult-to-treat (DTR) resistance category was also applied for Gram-negative bacteria, based on Kadri et al. [42], which correlates strongly with the clinical outcomes of infection. DTR can be described as a category within MDR isolates, where resistance to all first-line agents, i.e., all β-lactams, including carbapenems and β-lactamase inhibitor combinations, and fluoroquinolones is present.

### 4.5. Statistical Analyses

Descriptive statistical analysis (including means or medians with ranges and percentages to characterize data) was performed using Microsoft Excel 2013 (Redmond, WA, USA, Microsoft Corp.). R (version 4.0.1, R Foundation for Statistical Computing Vienna, Austria) program was used for data analysis and visualization.

### 4.6. Ethical Considerations

This study is part of the Antimicrobial Stewardship Programme of the University and has been approved by the Regional and Institutional Ethical Board. 

## 5. Conclusions

The frequent isolation of ESKAPE bacteria and the detected considerable acquired resistance among ED patients raise concern and indicate that these organisms are present/has been disseminated in the community as well. Understanding the local epidemiology of bacterial pathogens may provide benefits in establishing local empirical treatment protocols.

## Figures and Tables

**Table 1:** Epidemiology and distribution of ESKAPE pathogens during the study period.

Gram Stain	Bacterial Family/Genus/Species	Frequency (%)
Gram-positive *n* = 1032 (20.75%)	*Enterococcus* spp.	471 (9.47%)
	*Staphylococcus aureus*	561 (11.28%)
Gram-negative *n* = 3942 (79.25%)	*Stenotrophomonas maltophilia*	4 (0.08%)
	*Klebsiella* spp.	664 (13.35%)
	*Acinetobacter baumannii*	31 (0.62%)
	*Pseudomonas aeruginosa*	181 (3.64%)
	Enterobacterales*: Escherichia* spp.	2194 (44.11%)
	Enterobacterales: *Proteus* spp.	526 (10.57%)
	Enterobacterales: *Enterobacter* spp.	119 (2.39%)
	Enterobacterales:*Morganella* spp.	75 (1.51%)
	Enterobacterales: *Citrobacter* spp.	53 (1.07%)
	Enterobacterales: *Providencia* spp.	40 (0.8%)
	Enterobacterales: *Salmonella* spp.	33 (0.66%)
	Enterobacterales: *Serratia* spp.	22 (0.44%)
Total		4974 (100%)

**Table 2:** Distribution of ESKAPE isolates by clinical specimens.

Acro-nym	Bacterial Family/Genus/Species	Blood Culture (%)	Catheter-Specimen Urine (%)	Midstream Urine (%)	Wound or Abscess (%)	Others (%)	Total (%)
E	*Enterococcus* spp.	93 (19.7%)	191 (40.6%)	76 (16.1%)	48 (10.2%)	63 (13.4%)	471 (100%)
S	*Staphylococcus aureus*	252 (64.5%)	12 (3.1%)	6 (1.5%)	121 (30.9%)	0 (0%)	391 (100%)
	*Stenotrophomonas maltophilia*	(0%)	1 (0.6%)	(0%)	3 (1.7%)	170 (97.7%)	174 (100%)
K	*Klebsiella* spp.	212 (31.9%)	234 (35.2%)	118 (17.8%)	49 (7.4%)	51 (7.7%)	664 (100%)
A	*Acinetobacter baumannii*	6 (19.4%)	6 (19.4%)	(0%)	5 (16.1%)	14 (45.2%)	31 (100%)
P	*Pseudomonas aeruginosa*	47 (26%)	53 (29.3%)	16 (8.8%)	25 (13.8%)	40 (22.1%)	181 (100%)
E	Enterobacterales: *Citrobacter* spp.	13 (24.5%)	17 (32.1%)	7 (13.2%)	9 (17%)	7 (13.2%)	53 (100%)
	Enterobacterales: *Enterobacter* spp.	27 (22.7%)	30 (25.2%)	9 (7.6%)	24 (20.2%)	29 (24.4%)	119 (100%)
	Enterobacterales: *Escherichia* spp.	676 (30.8%)	823 (37.5%)	531 (24.2%)	75 (3.4%)	89 (4.1%)	2194 (100%)
	Enterobacterales: *Morganella morganii*	25 (33.3%)	11 (14.7%)	2 (2.7%)	21 (28%)	16 (21.3%)	75 (100%)
	Enterobacterales: *Proteus* spp.	116 (22.1%)	174 (33.1%)	44 (8.4%)	99 (18.8%)	93 (17.7%)	526 (100%)
	Enterobacterales: *Providencia* spp.	6 (15%)	15 (37.5%)	2 (5%)	8 (20%)	9 (22.5%)	40 (100%)
	Enterobacterales: *Salmonella* spp.	29 (87.9%)	3 (9.1%)	1 (3%)	0 (0%)	0 (0%)	33 (100%)
	Enterobacterales: *Serratia* spp.	5 (22.7%)	1 (4.5%)	2 (9.1%)	7 (31.8%)	7 (31.8%)	22 (100%)
Total		1507 (30.3%)	1571 (31.6%)	814 (16.4%)	494 (9.9%)	588 (11.8%)	4974 (100%)

Others = skin, cerebrospinal fluid, respiratory.

**Table 3:** Antibiotic resistance levels of ESKAPE isolates during the study period.

Bacterial Family/Genus/Species	Isolates (100%)	Wt (%)	UDR (%)	MDR (%)	DTR (%)	XDR (%)
*Enterococcus* spp.	471			18 (3.82%)		0 (0%)
*Staphylococcus aureus*	561			110 (19.61%)		0 (0%)
*Stenotrophomonas maltophilia*	4		0 (0%)	0 (0%)	0 (0%)	0 (0%)
*Klebsiella* spp.	664	390 (58.73%)	274 (41.27%)	182 (27.41%)	1 (0.15%)	0 (0%)
*Acinetobacter baumannii*	31	4 (12.90%)	27 (87.10%)	21 (67.74%)	13 (41.94%)	0 (0%)
*Pseudomonas aeruginosa*	181	117 (64.64%)	64 (35.36%)	24 (13.26%)	8 (4.42%)	1 (0.55%)
*Citrobacter* spp.	53	38 (71.70%)	15 (28.30%)	6 (11.32%)	0 (0%)	0 (0%)
*Enterobacter* spp.	119	79 (66.39%)	40 (33.61%)	14 (11.76%)	0 (0%)	0 (0%)
*Escherichia* spp.	2194	891 (40.61%)	1303 (59.39%)	572 (26.07%)	0 (0%)	0 (0%)
*Morganellas* spp.	75	12 (16%)	63 (84.00%)	14 (18.67%)	0 (0%)	0 (0%)
*Proteus* spp.	526	129 (24.52%)	397 (75.48%)	214 (40.68%)	1 (0.19%)	0 (0%)
*Providencia* spp.	40	14 (35%)	26 (65.00%)	5 (12.50%)	0 (0%)	0 (0%)
*Salmonella* spp.	33	28 (84.85%)	5 (15.15%)	0 (0%)	0 (0%)	0 (0%)
*Serratia* spp.	22	13 (59.09%)	9 (40.91%)	3 (13.64%)	0 (0%)	0 (0%)
Total	4974	2751 (55.31%)	2223 (44.69%)	1183 (23.78%)	23 (0.46%)	1 (0.02%)

Wt: wild type, UDR: usual drug resistance, MDR: multi-drug resistance, DTR: difficult to treat resistance, XDR: extensively drug-resistant.

**Table 4:** Rates of susceptibility and multi-drug resistance for Gram-positive ESKAPE isolates.

	OXA	AMP	CC	CIP *	ERY	GM	LNZ	TEC	VA	TGC	SXT	MDR
*Enterococcus faecalis*	IR	100.0	IR	56.8	IR	IR	99.1	100.0	100.0	99.3	IR	0.7
*Enterococcus faecium*	IR	IR	IR	7.1	IR	IR	100.0	78.8	66.7	100.0	IR	45.5
*Staphylococcus aureus*	83.1	IR	79.4	80.7	78.0	98.6	100.0	100.0	100.0	100.0	98.6	19.6
*MSSA*	-	IR	89.2	92.8	88.2	99.4	100.0	99.8	100.0	100.0	98.7	3.2
*MRSA*	-	IR	30.9	22.1	27.7	94.7	100.0	100.0	100.0	100.0	97.9	100.0

*: relevant only for urinary specimens, IR = intrinsic resistance, OXA = oxacillin, AMP = ampicillin, CC = clindamycin; CIP = ciprofloxacin; ERY = erythromicin; GM = gentamicin; LNZ = linezolid; TEC = teicoplanin; VA = vancomycin, TGC = tigecyclin; SXT = sumetrolim; MSSA = methicillin sensitive *S. aureus*; MRSA = methicillin-resistant *S. aureus.* Green: susceptibility rate over 90%, may be prescribed empirically even in severe infections, Yellow: susceptibility rate over 80%, may be prescribed empirically except severe infection, Red: should not be prescribed empirically. If an isolate is susceptible in high rate to certain antibiotics (e.g., MSSA to ciprofloxacin or to gentamicin) it does not mean that the antibiotic is a recommended agent. If an isolate is susceptible in low rate to certain antibiotics (e.g., *Enterococcus faecium* to vancomycin) it does not mean that the agent cannot be used in selected cases.

**Table 5:** Rates of susceptibility and multi-drug resistance for Gram-negative ESKAPE isolates.

	AMP	AMC	CXM	CTX	CAX	CAZ	FEP	TZP	ETP	MEM	CIP	AK	GM	SXT	MDR
*Klebsiella aerogenes*	0.0	IR	66.7	75.0	80.6	80.6	85.7	80.0	100.0	100.0	87.1	100.0	96.8	93.5	22.6
*Klebsiella oxytoca*	0.0	91.1	96.8	98.0	97.5	97.5	98.0	98.0	100.0	100.0	100.0	98.0	100.0	98.7	2.53
*Klebsiella pneumoniae*	0.0	67.5	67.2	73.0	69.4	69.4	76.6	76.1	99.8	99.8	62.8	79.3	79.1	65.5	32.6
*Klebsiella variicola*	0.0	100.0	100.0	100.0	100.0	100.0	100.0	100.0	100.0	100.0	100.0	100.0	100.0	100.0	0.0
*Acinetobacter baumannii*	NT	IR	NT	NT	IR	NT	NT	NT	IR	55.2	13.8	37.9	40.7	21.4	65.5
*Pseudomonas aeruginosa*	NT	IR	NT	NT	IR	90.6	91.7	87.2	IR	85.6	77.9	96.7	90.1	NT	13.3
*Citrobacter freundii*	0.0	IR	20.0	82.4	71.4	71.4	82.4	82.4	100.0	100.0	90.5	100.0	100.0	100.0	19
*Citrobacter koseri*	0.0	81.5	77.3	100.0	100.0	100.0	100.0	85.7	100.0	100.0	100.0	100.0	100.0	100.0	3.7
*Enterobacter cloacae*	0.0	IR	38.8	84.4	83.0	83.0	84.4	84.1	100.0	100.0	93.3	98.4	98.9	93.2	11.2
*Escherichia coli*	48.5	81.8	85.4	85.8	86.1	86.2	86.7	85.0	100.0	100.0	67.9	89.2	90.3	72.7	26.1
*Morganella morganii*	0.0	IR	0.0	93.3	90.3	90.3	93.3	90.0	100.0	100.0	90.3	91.5	91.7	72.2	18.1
*Proteus mirabilis*	40.4	62.3	63.0	71.7	69.1	69.1	75.5	74.7	99.8	100.0	57.7	75.7	77.2	32.1	42.7
*Proteus vulgaris*	0.0	40.0	0.0	95.5	91.4	91.4	95.5	95.5	100.0	100.0	85.7	95.5	91.4	65.7	14.3
*Providencia stuartii*	0.0	IR	31.6	93.3	96.6	96.6	92.9	92.9	100.0	100.0	72.4	36.4	28.6	29.6	17.2
*Salmonella* spp.	90.6	100.0	100.0	100.0	100.0	100.0	100.0	100.0	100.0	100.0	96.0	100.0	100.0	96.9	0.0
*Enterobacterales*	34.6	70.4	76.0	83.1	82.3	82.3	84.9	83.5	99.9	100.0	69.4	86.1	87.5	68.5	27.1
*Enterobacterales non ESBL*									100.0	100.0	82.2	93.5	93.8	77.1	11.8
*Enterobacterales ESBL*									99.7	99.8	9.6	46.8	58.6	27.9	98.5

Only bacteria with minimum 20 separate isolation are shown in the Table. IR: intrinsic resistance, AMC = co-amoxiclav; CTX = cefotaxime; CAX = ceftriaxone, CAZ = ceftazidime; FEP = cefepime; TZP = piperacilllin tazobactam, ETP = ertapenem; MEM = meropenem; CIP = ciprofloxacin; AK = amikacin; GM = gentamicin, COL = colistin, MDR = multi-drug resistance, Green: susceptibility rate over 90%, may be prescribed empirically even in severe infections, Yellow: susceptibility rate over 80%, may be prescribed empirically except severe infection, Red: should not be prescribed empirically. If an isolate is susceptible in high rate to certain antibiotics it does not mean that the antibiotic is a recommended agent. If an isolate is susceptible in low rate to certain antibiotics (e.g., *Escherichia coli* to ciprofloxacin) it does not mean that the agent cannot be used in selected case.

## Data Availability

All data generated during the study is presented in this paper.
